# Supplementary material for: Acceptability of the Wulira app in assessing occupational hearing loss among workers in a steel and iron manufacturing industry
Source: PLoS One. 2022 Oct 7;17(10):e0266858. doi: 10.1371/journal.pone.0266858 (PMC9543627; doi:10.1371/journal.pone.0266858)
Supplement: S1 File — (DOCX) [file pone.0266858.s001.docx]

**FGDs Guide tool.**

**ACCEPTABILITY OF THE WULIRA APP IN ASSESSING OCCUPATIONAL HEARING LOSS AMONG WORKERS IN A STEEL AND IRON INDUSTRY**.

I am **(name)** and on behalf of Dr. Immaculate Atukunda, a Lecturer at Makerere University College of Health Sciences, I am conducting this FGD to assess the acceptability of using Wulira App to screen for hearing loss among industry workers. You have been asked to participate in this exercise because you work in a steel manufacturing industry and at a risk of developing occupational hearing loss. This exercise will take approximately 30- 45 minutes.

**Focus group Discussions Guide**

- 1. In your opinion, what is occupational hearing loss?
  2. Have you undergone testing for hearing loss? If so, where did you go and what did they use to test you for hearing loss?
  3. Was the Wulira app used to test your hearing ability? If it was used, how did you find the exercise? Would you mind telling me how the exercise went? (Affective attitude)
  4. Tell me about the duration of the screening exercise using Wulira App. How long did it take to start and end? Was there any inconvenience you felt during this exercise? (Burden)
  5. Do you prefer to use Wulira App or other tools to screen for hearing loss? And Why would you prefer that tool? (belief)
  6. Tell me what took place during hearing ability testing using Wulira App? (Intervention coherence)
  7. Did you incur any cost during the testing exercise? If you did, how much was it and what would you have used the money for? If you did not, what were you going to use the money for or what were you going to be engaged in during this time? (opportunity cost)
  8. What do you think about the success of using Wulira App to screen hearing loss? Was it successful? If it was, why do you this so? (Perceived Effectives)
  9. Will you be able to do frequent screening for hearing performance? Why do you think it is necessary? (Self- efficacy)

*Thank you for participating.*

**INTERVIEW GUIDE**

1. In your opinion, what is occupational hearing loss?
2. Do you encourage your workers to test for hearing loss? If so, where did they go and what do they use to test for hearing loss? How often do you ask then to go for hearing performance testing?
3. Was the Wulira app used to test the hearing performance for your workers? If it was used, how was the testing exercise organized? How did it go? (Affective attitude)
4. Tell me about the duration of the screening exercise using Wulira App. How long did it take to start and end? Did the screening exercise affect the work schedule of your staff during this exercise? (Burden)
5. What is your preference? Would you recommend the Wulira App or other tools to test hearing performance? And Why would you prefer that tool? (belief)
6. In simple term, could you describe how testing for hearing performance was done using Wulira App? (Intervention coherence)
7. Do you think participating in the exercise was beneficial to the company and its employees? If yes, how was it beneficial? If no, was there any activity your employees should have been engaged in? (opportunity cost)
8. What are your thoughts on the success of using Wulira App to test for hearing performance? (Perceived Effectives)
9. Why is it necessary to do frequent screening for hearing loss among industrial workers? Will you company opt for Wulira App as a screening tool in future? (Self- efficacy)

*Thank you for participating.*
