# Supplementary material for: Acceptability of the Wulira app in assessing occupational hearing loss among workers in a steel and iron manufacturing industry
Source: PLoS One. 2022 Oct 7;17(10):e0266858. doi: 10.1371/journal.pone.0266858 (PMC9543627; doi:10.1371/journal.pone.0266858)
Supplement: Attachment — Submitted filename: Responses to Reviewer.docx [file pone.0266858.s004.docx]

*Generally, the authors appropriated response to my comments and suggestions. However, some changes are needed before publication:*

*- I am not a native English speaker but I believe that your manuscript could benefit by English language professional service.*

**Authors’ Response:**

Thanks for the suggestion. The manuscript has been proof read and has been remarkably improved regarding the grammar.

*- Write the aim of the study at past tense.*

**Authors’ Response:**

Thanks for this observation. This has been updated as suggested.

*- Some duplicated text exists in the revised manuscript (e.g. Affective attitude).*

**Authors’ Response:**

Thanks for this observation. We have proof read the manuscript. The updated manuscript has no duplicated text.

*- Line 230-231 belongs to the methods section.*

**Authors’ Response:**

Lines 230-231 have statements in the result section.

We believe you meant lines 440-441 in the limitation section. We acknowledge this statement should be in the methodology, however it was included here to explain the potential bias of our study and how we addressed this.

*- Do not start a sentence with a number.*

**Authors’ Response:**

Thanks for the suggestion. The sentence that was started with a number has been corrected as suggested.
